# Subthreshold Autism Spectrum in a Patient with Anorexia Nervosa and Behçet's Syndrome

**DOI:** 10.1155/2020/6703979

**Published:** 2020-06-15

**Authors:** L. Dell'Osso, B. Carpita, I. M. Cremone, F. Mucci, A. Salerni, D. Marazziti, C. Carmassi, C. Gesi

**Affiliations:** Department of Clinical and Experimental Medicine, University of Pisa, Pisa, Italy

## Abstract

Recently, increasing research stressed the presence of subthreshold autistic traits in patients with other psychiatric conditions. In this framework, a significant relationship between anorexia nervosa (AN) and the autism spectrum has been frequently reported, in particular among female samples, to the point that AN has been hypothesized to be a female phenotype of autism spectrum disorder (ASD). On the other hand, among subjects with ASD has been reported a higher prevalence of immune diseases and altered immune functions. While these reports seem to support an association between neurodevelopmental and immune system alterations in ASD, the relationship between the immune system and the broader autism spectrum, including its subthreshold manifestations, remains poorly investigated. In this report, we described the presence of autistic traits in a male inpatient with AN and separation anxiety disorder, who also show a diagnosis of Behçet's syndrome (BS). This case seems to further stress the association between AN and the autism spectrum, which may not be limited to the female gender. Moreover, it further suggests a deeper link between neurodevelopmental and immune system alterations. Implications are discussed in light of the more recent neurobiological and psychopathological hypothesis about the autism spectrum.

## 1. Introduction

Autism spectrum disorder (ASD) is characterized by a cluster of symptoms (impairment in communication and social interaction, restricted pattern of interests, and repetitive behaviors) associated with a neurodevelopmental alteration, usually manifesting in early childhood [1]. Recently, increasing interest has been paid to investigating the presence of subthreshold autistic traits, providing support to the hypothesis that a subthreshold autism spectrum may represent a dimension spanning across several mental disorders [1]. The interest in investigating autistic traits lies in the fact that a growing number of studies are stressing their role as a vulnerability factor towards the development of different kinds of psychiatric disorders, as well as suicidal thoughts and behaviors [1–9]. Moreover, milder forms of ASD, without intellectual impairment and with less evident social difficulties, may often remain underdiagnosed, coming to clinical attention only during adult life, when patients develop other disorders in comorbidity [1, 2]. In this framework, a significant relationship of anorexia nervosa (AN) with both ASD and autistic traits has been reported in the literature [10, 11]. Although ASD has been considered a disorder with a strikingly higher prevalence among males, recently several authors questioned this data [12, 13]. Evidence from literature seems to point out that gender differences in ASD prevalence may be related to a male bias in defining ASD criteria [10–13]. ASD is likely to be more prevalent than previously expected among females, although in this group, it would manifest with different kinds of symptoms [10–13]. Firstly, females with ASD frequently show a higher ability to cope with social difficulties by the use of camouflaging strategies, such as imitating others' behaviours, and, as a consequence, milder forms of ASD in females would remain more frequently undiagnosed [14]. Moreover, females with ASD often show a different pattern of restricted interests, such as spending time with animals, fictions, and also focusing on food and diet [10–13, 15]. Since the ‘80s, the association of ASD or subthreshold autistic traits with AN has gained increasing interest in the scientific literature. After the first studies which reported a higher presence of AN among female relatives of ASD patients, several researches were conducted on the prevalence of autistic-like features among females with AN, also with a longitudinal design, confirming the presence of significant overlap between the autism spectrum and AN [10–13]. In light of these findings, some authors also hypothesized that AN should be considered as a specific presentation of ASD, which would be more frequent among females [10–13]. However, most of the studies about AN and the autism spectrum (ASD or subthreshold autistic traits) were conducted in samples composed almost exclusively by females, and no study specifically focused on the relationship between ASD and AN among males [12, 13]. In addition, few studies investigated gender differences in clinical presentations of AN, mostly reporting only a higher tendency towards a chronic course among females, while no significant difference was found for other clinical features [16]. The presence of a lower focus on thinness and a higher focus on other features (such as large shoulders and narrow waist) among males was not confirmed by all the studies [17].

On the other hand, among subjects with ASD has been reported a higher prevalence of immune diseases, as well as findings of altered immune functions [18, 19]. Some studies highlighted also, among mothers of subjects with ASD, the presence of autoantibodies directed against fetal brain tissue [20, 21]. While these reports seem to support an association between neurodevelopment alterations and immune system dysfunctions in ASD, the relationship between the immune system and the broader autism spectrum, including its subthreshold manifestations, has been poorly investigated [19]. In this case presentation, we aim to report and discuss the presence of autistic traits in a male patient with AN and Behçet's syndrome (BS).

## 2. Case Presentation

Mr. XY is a 33-year-old man who was referred to the psychiatric service by his rheumatologist for a severe weight loss associated with complete food refusal. He was admitted to the psychiatric inpatient unit, and detailed information about the case history was collected.

Mr. XY was first diagnosed with a BS in 2003, over twelve years before the first psychiatric admission. BS is a disease featuring a chronic vasculitis with a multisystemic involvement of both arteries and veins [22]. Orogenital and ocular lesions are common in BS, but it may show a wide range of clinical manifestations, including involvement of the central nervous system (CNS) [22, 23].

BS is supposed to have a strong genetic vulnerability and, although its pathogenesis is still not clear, it was associated with an alteration of the immune and inflammatory response [22, 23]. Soon after, Mr. XY underwent a genetic examination, showing a HLA-B51 carrier status (an allele associated with the presence of BS) [22]; moreover, a brain magnetic resonance imaging (MRI) highlighted a diffuse signal intensity alteration of the right cerebellar hemisphere. He was first prescribed glucocorticoids (often employed in the treatment of the acute involvement of the CNS in BS) and colchicine, an alkaloid with anti-inflammatory and immune-modulatory properties used with beneficial effects in different kinds of diseases, from BS from gout [23, 24]. It was also prescribed cyclosporine, an immunosuppressive agent that interferes with the transcription of several cytokines [25]; however, cyclosporine intake was discontinued by Mr. XY in a few months because of gastrointestinal side effects. Since two years from the first diagnosis, he had started to show signs of neuromuscular damage (including tremors, muscle weakness with recurrent falls, dyspnea with reduced exercise tolerance, and evidence of restrictive ventilatory defect with no diffusion impairment). During 2009, he reported recurrent headaches, and as a consequence, a new brain MRI was performed, showing a cavernous angioma located in the right cerebellar hemisphere. Two years later, a further MRI showed a second cavernous angioma in the right ventromedial prefrontal cortex and a venous dysplasia in the right cerebellar hemisphere. A detailed psychiatric history was also obtained. Mr. XY was the first of four children. No family history of mental disorders was reported. He recounted himself as a child and adolescent with pronounced anxiety traits: when he was between four and nine years old, he was quite worried of being separated from his mother, showing a lack of independence in several contexts, such as sleeping, socializing, and attending school. During adolescence and early adulthood, he continued to be overly worried about his parents' sake and to suffer from anxiety symptoms, avoiding social contexts. He never enjoyed practicing sports and he remembers himself as having always been thin and weak. After high school, he started to work as a religion teacher. He had only one stable relationship, lasting about four years, with a woman nine years older than him; he had no children. He described himself as a quiet person, very introspective, and ruminative. Besides his job, he devoted himself to political interests, taking part in the council of the small town where he lived. He never smoked or used illicit drugs. About ten months before the current admission, Mr. XY was shocked by the sudden death of a member of the town council, 30 years older than him, whom the patient remembers as a close friend. Shortly after, he started to be very focused on diet, restricting food intake and selecting only low-calorie food. However, spontaneously, in a few weeks, he regained a normal eating behavior and sufficient social and work adjustment. Despite that, he continued to show intense rumination over his friend's death and started again to fear that some untoward events could happen to his parents. One month before the first psychiatric admission, Mr. XY showed another episode of food restriction, following the break up of his relationship with his girlfriend. He suddenly developed depressive mood, feelings of hopelessness, inner tension, outbursts of anger, and rumination with difficulties to fall asleep. Despite having several talks with his ex-girlfriend, he felt he could not understand the reasons why she broke up with him and started a calorie-restricted diet, feeling that taking control of his body shape could help him to boost his self-esteem and hopefully to bring back his ex-girlfriend. When he saw his rheumatologist, who had treated him for more than 10 years, he had lost about 8 kg in a month and his BMI was around 18.4, such that he was referred to the psychiatric team. He was admitted voluntarily to the psychiatric inpatient unit. On admission, Mr. XY appeared very skinny, moderately unkempt, and slow at movement. His eyes moved little, staring with a plane face at the clinician's eyes. His speech was slow and circumstantial; answers were given in monosyllables, with a monotone voice. He was strongly worried with his body weight and with food calorie content, completely refusing to eat, such that nasogastric feeding was needed.

Mr. XY was diagnosed as having a partial form of posttraumatic stress disorder (PTSD), a separation anxiety disorder, and AN. However, during his hospital stay, a peculiar social functioning was progressively observed. Mr. XY continued to show a staring gaze, approaching people in an awkward way, without showing social awareness, as if he disregarded other people's feelings. Even when talking about emotionally charged situations, he used a matter-of-fact tone. Despite stating that he was desperate, he appeared calm, being always complacent towards anyone. When asked about his feelings, he found it very hard to express them, rationalizing and intellectualizing his difficulties.

Taking into account these traits, which suggested autistic-like impairments in social communication and in socioemotional reciprocity, a deeper evaluation was conducted. First, Mr. XY's mother was asked to answer some questions, revealing that Mr. XY had been referred to a psychologist during elementary school for anxiety symptoms, but also because he used to be easily distracted by sounds and other stimuli during classes. Moreover, she reported that his classmates often picked on him for his social awkwardness and his overinvolvement in mechanical gadgets.

In addition, Mr. XY agreed to complete the short (14-Item) version of the Ritvo Autism and Asperger Diagnostic Scale-Revised (RAADS-R) [26], a self-report screening questionnaire for ASD, retrieving a score of 11 (cut-off score 14); he fulfilled also the self-report Autism Spectrum Quotient (AQ) [27] and the Adult Autism Subthreshold Spectrum (AdAS Spectrum) [28], both providing a dimensional measure of autistic traits, reporting a score of 22 and 51, respectively. He also gave written informed consent for the publication of his case.

When discharging from the hospital, the initial diagnoses of partial PTSD, separation anxiety disorder, and AN were confirmed. However, the psychiatric team agreed that a subthreshold autism spectrum was also present and could have shaped the clinical picture of Mr. XY.

## 3. Discussion

In light of the most recent literature, the autistic-like traits found in Mr. XY allow a more accurate description of the patient's phenotype, and in addition, they may also offer better insight on several aspects of his clinical presentation.

First of all, subjects with ASD are known to show significant difficulties in adjusting to life events, eventually showing an increased vulnerability towards the development of suicidal behaviors in the context of an adjustment disorder [29]. Moreover, autistic traits have been reported to increase the likelihood of developing posttraumatic symptoms, leading to hypothesize that some autistic dimensions might raise the risk of a broad range of trauma and stress-related disorders [5, 6, 30, 31].

On the other hand, despite no studies have so far investigated the relationship between separation anxiety and autistic traits, subjects with ASD often experience symptoms of separation anxiety [32] that are in turn tightly interwoven with pathological reactions to grief [33]. According to these observations, it might be hypothesized that not only full-blown autism but also high levels of autistic traits would be associated with significant separation sensitivity: this latter may have contributed to trigger the complex reaction to separation and loss exhibited by Mr. XY.

Furthermore, some remarks can be made about the relationship between Mr. XY's autistic traits and AN. The existing literature, mainly based on samples of females with AN, consistently shows that AN is associated with certain premorbid traits, such as perfectionism, inhibition, anxiety, and rigidity [34, 35], while the scant literature on AN among males reports similar traits in anorexic males as well [16, 17]. More recently, specific attention has been devoted to the rigid eating habits showed by women with AN, which resemble the narrow interests and repetitive behavior of autistic people, and to the deficits in theory of mind and social intelligence that seem to be common in both AN and ASD [36–38]. As stated above, AN has been hypothesized to be a specific ASD phenotype (which would be more frequently, but possibly not exclusively, associated with female gender), characterized by restricted interest and repetitive behaviors focused on food and diet [12, 13]. On this basis, autistic dimensions have been investigated mostly among AN females, who showed higher levels of autistic traits compared to healthy control subjects [10, 11]. However, while female AN has already been linked to high levels of autistic traits, no study specifically focused on investigating the relationship between AN and ASD, or subthreshold autistic traits, in male samples. The reported case of an adult male with AN and autistic-like features may lead to hypothesize that autism spectrum and AN would be associated also among males and that atypical presentations of autistic traits would not be limited to the female gender. Mr. XY, in fact, reported a RAADS-14 score slightly under the threshold for a possible diagnosis of ASD. Accordingly, he did not endorse all criteria needed for a clinical diagnosis of ASD according to DSM-5 guidelines. However, he reported a score of 22 on the AQ, an instrument developed to quantify autistic traits in both clinical and nonclinical samples. Interestingly, Mr. XY's score of 22 is quite close to the mean score of about 21 found by Baron-Cohen and colleagues among AN female patients [39] and far above mean scores reported among healthy controls [40, 41]. Although a significant disproportion between males and females in AN prevalence is usually reported, gender has been actually reported to have little effect on the clinical features of AN, with the only exception of a more chronic course among females [16]. As previously reported in female samples, the case of Mr. XY shows the presence of autistic traits in a male inpatient with AN, confirming that similarities in the clinical presentation of AN between males and females might outweigh the differences, being linked to the autism spectrum in both sexes. However, given the unique nature of this case in literature, and the rarity of AN diagnosis among males, findings from this case are of limited generalizability, and further studies are requested to clarify the relationship between autism spectrum and AN in light of gender differences: deepening the knowledge in this field may lead to a better understanding of eventual gender biases in both autism spectrum and eating disorders.

In this case, it is noteworthy that Mr. XY shows not only AN but also a higher vulnerability towards developing psychiatric symptoms after adjustment to separation and grief. Moreover, he also showed social phobic traits, another element that has been associated with ASD and female gender [41, 42]. In particular, social anxiety (which features, as ASD, an impairment in the social brain) [43, 44] has been hypothesized to be higher in subjects with autistic traits or milder forms of ASD, who would be more aware of their social difficulties compared to patients with more severe forms of the disorder [41].

This clinical picture should be globally considered in light of the most recent hypotheses in the field of psychopathology, which suggest the possible presence of a neurodevelopmental alteration at the basis of different kinds of psychiatric conditions [45–47]. According to this conceptualization, while the specific severity and timing of the neurodevelopmental alteration and its interaction with the environment may determine different psychopathological trajectories, a common feature of this “neurodevelopmental continuum” would be a higher vulnerability towards traumatic or stressful events during lifetime [45–47]. This perspective may shed more light on the altered, often autistic-like, premorbid functioning reported in psychiatric patients suffering from different kinds of disorders (AN, mood disorders, and schizophrenia) [13, 48–50] as is the case of Mr. XY. Finally, the occurrence of autistic traits in a subject suffering from BS is of specific interest. In both ASD and BS, involvement of the immune system has been hypothesized. For example, dysregulation of immune function and neuroinflammation, as well as the presence of maternal autoantibodies directed against fetal brain tissue, have been involved in the pathophysiology of ASD [19, 21]. Recently, some authors highlighted the potential role of maternal antibodies against stress-induced phosphoprotein 1 (STIP1) [51], a ligand of prion protein found in the developing brain that has been shown to mediate neurogenesis in cultured hippocampal neurons [52, 53]. Intriguingly, previous data also described the presence of autoantibodies to STIP1 in the blood and CSF of patients with neuro-Behçet's disease [54]. A precocious involvement of the central nervous system, usually occurring in 4 to 49% of BS sufferers after more than 10 years of disease [55], clearly emerges also from Mr. XY's clinical history, allowing speculating that both Mr. XY's autistic subthreshold spectrum and neuro-Behçet symptoms might share a common, autoimmune physiopathology. Globally, this case adds to previous literature that stressed a deep intertwining between the immune system and neurodevelopmental alterations. The possible link between ASD and autoimmunity has been a recurrent topic since 1970, when authors highlighted the presence of immune aberrations and altered immune responses in children with ASD, including a higher prevalence of allergies [56]. In the last decades, increasing literature highlighted several kinds of immune system alterations, such as altered levels of cytokines, in patients with ASD [57]. The authors suggested the presence of shared genetic underpinnings between ASD and autoimmune diseases, which would interact with early environmental factors, in particular during intrauterine life, in shaping neuroimmune patterns [19, 21]. On the other hand, also AN has been linked with the presence of immune system alterations: while some authors stressed the presence of a higher vulnerability of patients with autoimmune diseases towards the development of AN, pointing out a possible role of the immune system in the pathogenesis of eating disorders [58], others highlighted the presence of immune system dysregulation in patients with AN, hypothesizing that it may be caused by the altered nutritional status [59]. In this framework, recent researches on the role of gut microbiota in both immune and psychiatric disorders have further broadened the perspectives [19]. Microbiota composition has been reported to be altered in both ASD and AN, and several authors hypothesized a role of the so-called microbiota-gut-brain axis in the pathophysiology of these disorders [19, 60]. It is noteworthy that the first investigations on the link between microbiota and ASD were conducted after the frequent observations of gastrointestinal symptoms and/or disordered eating habits in ASD children, further stressing the link between ASD and AN [19]. Microbiota is also known for its crucial role in modulating the immune response, including cytokine levels, exerting proinflammatory or anti-inflammatory activities depending on its compositions [19]. Many authors hypothesized a possible biunivocal influence between microbiota and the CNS through the immune system [19, 61]. The case of Mr. XY seems in line with these hypotheses, showing the presence, at the same time, of autistic symptoms, AN, and BS, a disease linked to altered immune response. However, this case is, at the same time, quite unique in the scientific literature, where the research on ASD and AN mostly focused on females. Moreover, while, as stated above, both autism spectrum and AN were reported to be associated with immune system alterations, scant literature focused on the concomitant occurrence of these three conditions. A recent review further remarked the link between ASD, AN, specific immune alterations, and microbiota profiles, suggesting that future researches in the field of immunopsychiatry should focus on mucosal immunity [61]. Within an integrative framework between central and peripheral systems, more clinical research is needed to clarify the link between neurodevelopment, immune system, and eating disorders.
